# Neuroprotective effects of chloroform and aqueous fractions of noni juice against t-Butyl hydroperoxide-induced oxidative damage in SH-SY5Y cells

**DOI:** 10.29219/fnr.v62.1605

**Published:** 2018-12-19

**Authors:** Jianguo Chen, Xue Shi, Yang Chen, Hanqiao Liang, Chi Cheng, Qiyang He

**Affiliations:** 1 Inner Mongolia Mengniu Diary Industry Group Co, Ltd, China; 2 Institute of Medicinal Biotechnology, Peking Union Medical College and Chinese Academy of Medical Sciences, Beijing, China; 3 China Center of Industrial Culture Collection, China National Research Institute of Food and Fermentation Industries, Beijing, China

**Keywords:** noni juice, neuroprotective, oxidative stress, Nrf2, apoptosis

## Abstract

Oxidative stress is more likely to cause damage to neuronal cells and mediates some neurodegenerative disorders. It is well known that natural antioxidants can prevent oxidative stress damage and become a potential therapeutic strategy. Noni juice obtained from the fruit of the tree *Morinda citrifolia*, as a folk medicine, has been used for over two thousand years. In the current study, the neuroprotective effect and mechanism of noni juice extracts against tert-Butyl hydroperoxide (TBHP)-induced SH-SY5Y cell damage were investigated. The results demonstrated that chloroform fraction (CF) and aqueous fraction (AF) of noni juice protected SH-SY5Y cells against TBHP-induced oxidative stress and the associated apoptosis effectively. CF and AF treatment significantly weakened the TBHP-induced cytotoxicity, reactive oxygen species generation, mitochondrial membrane depolarization, and apoptotic features. CF and AF restored cellular antioxidant enzyme activity; upregulated expression of heme oxygenase-1, catalase, and superoxide dismutase-1; and increased the nuclear accumulation of nuclear factor-erythroid 2 related factor 2 (Nrf2). The antioxidant and neuroprotection potential of CF may account for its high total phenolic and flavonoid content, while AF may be rich in polysaccharides. These results suggest that CF and AF exhibit antioxidant defense through the upregulation of Nrf2 along with endogenous antioxidants and reduce apoptosis via inhibiting the mitochondrial pathway to protect SH-SY5Y cells damaged by TBHP. CF and AF might be developed as agents for neurodegeneration prevention or therapy.

Compared with other organs, lower levels of antioxidant enzymes, higher metabolic activity and higher content of polyunsaturated fatty acids in the brain, neurons are more susceptible to oxidative stress (1, 2). The imbalance between oxidants and antioxidants may destroy the redox signal and lead to oxidative stress. Excessive oxidative stress can cause oxidative damage in biomolecules such as DNA, proteins, lipids, and carbohydrates. Many neurodegenerative diseases, such as Alzheimer’s disease, Parkinson’s disease, Huntington’s disease, and amyotrophic lateral sclerosis, may be caused by neuronal damage resulting from intracellular oxidative stress (3–8). Increasing evidence suggests that natural antioxidants may attenuate neurotoxicity and play important roles in prevention and treatment of oxidative stress-induced neurodegenerative diseases (9–12).

Herbal extracts rich in antioxidants, as traditional medications, have potent antioxidant activity and prevent neurodegenerative diseases caused by oxidative stress. *Morinda citrifolia* L. is commonly known as ‘noni’ and has been extensively used as a folk remedy for 2000 years in Polynesia, Australia, Southeast Asia, and Hawaii. Noni juice, obtained from the fruit of *M. citrifolia*, is reported to have many bioactive phytochemical constituents, such as glycosides, iridoids, anthraquinones, flavonoids, phenolic acid, and coumarins (13–15). It has been proven that noni juice exhibits many pharmacological properties, including antioxidant, anti-inflammatory, and antitumor effects (16–19). Therefore, it is suggested that the antioxidant and anti-inflammatory properties of noni juice can provide a protective effect against oxidative stress-induced neurodegenerative diseases.

Although protective effects of noni juice against scopolamine, ß-amyloid, and streptozotocin-induced memory impairment caused by focal ischemia and rotenone in animals have been reported (20–21), there is no data available on the neuromodulatory potential and molecular mechanism of noni juice or its extracts on neuronal cell damage. Thus, for the first time, we investigated the neuroprotection and potential mechanism of noni juice extracts against tert-Butyl hydroperoxide (TBHP)-induced neurotoxicity in SH-SY5Y cells.

## Materials and methods

### Preparation of noni juice extracts

Noni juice (10 liters) was purchased from Noni Biological Engineering Development (Hainan, China). Noni juice was partitioned successively with chloroform, ethyl acetate, and n-butanol. Each fraction was evaporated in a vacuum to obtain a chloroform fraction (CF, 3.3g), an ethyl acetate fraction (EF, 5.3g), an n-butanol fraction (BF, 60.7g), and an aqueous fraction (AF, 64.8g).

### Chemicals and reagents

TBHP and Rhodamine 123 were purchased from Sigma-Aldrich (St. Louis, MO, USA). An annexin V/propidium iodide (PI) double staining kit was purchased from Nanjing KeyGen Biotech Co., Ltd. (Nanjing, China). Catalase (CAT), superoxide dismutase-1 (SOD-1), glutathione peroxidase (GPx), and glutathione reductase (GR) kits were obtained from Nanjing Jiancheng Bioengineering Institute (Nanjing, China). A Cell Counting Kit-8 was purchased from Biotool (Shanghai, China). 5-(and-6)-carboxy-2’,7’-difluorodihydrofluorescein diacetate (H2DFFDA) was obtained from Invitrogen (Carlsbad, CA, USA).

### Animals

Specific pathogen-free Sprague-Dawley rats were used for the acute oral toxicity tests. Male and female rats (3-week-old) were provided by the Institute of Experimental Animals, Chinese Academy of Medical Sciences (Beijing, China). Animals were housed in the animal house with a controlled temperature of 24 μ 1°C, relative humidity of 60 μ 5%, and a 12-hr light–dark cycle.

### Cell culture

The SH-SY5Y human neuroblastoma cell line was purchased from the American Type Culture Collection (Rockville, MD, USA). The cells were cultured in Dulbecco’s Modified Eagle Medium/Ham’s F-12 (DMEM/F12) supplemented with 10% fetal bovine serum (Gibco, Australia) and maintained at 37°C in a humidified atmosphere with 5% CO_2_.

### Acute oral toxicity

Ten liters of noni juice was freeze-dried to obtain 69.4 g of powder. The noni juice powder was dissolved in distilled water at 375 mg/mL. Toxicity of the noni juice was assessed after a single oral administration in rats. The rats were randomly divided into two groups, each group with five males and five females. The rats were administered noni juice powder at 15,000 mg/kg body weight (BW) or the vehicle control (distilled water). Treatments (20 mL/kg BW) were administered a single dose by oral gavage. The single dose administration on the morning of Day 1 was followed by a 14-day observation period in which mortality or signs of morbidity were recorded for each animal. Animals were weighed immediately on Day 1 and again on Day 15.

### Bacterial reverse mutation test

Noni juice powder was assessed by two independent experiments for its potential to induce reverse mutation by the Ames test as described by Dillon GP (22). Genotoxicity was measured in *Salmonella typhimurium* strains TA 97, TA 98, TA 100, and TA 102. NJP was administered at 8, 40, 200, 1,000, and 5,000 μg/plate. All mutagenicity testing was performed in the presence or absence of the postmitochondrial fraction of liver homogenates (S9) from rats pretreated with Aroclor 1254.

### Determination of total phenolic, flavonoid, and polysaccharide contents

The total phenolic (TPC) and flavonoid contents were determined according to the method of Wu CR et al. (23). The TPC contained in each sample was expressed as milligrams of gallic acid equivalents (GAE) and flavonoid amount was expressed as milligrams of rutin equivalents (RE). Polysaccharide content was determined by a spectrophotometric method at 485 nm, modified from the method described by Li et al. (24). The amount of polysaccharide contained in each sample was expressed as milligrams of D-glucose equivalents (GE).

### Cytotoxicity of noni juice extracts and TBHP

The Cell Counting Kit-8 assay was used to assess cell viability. SH-SY5Y cells were seeded into 96-well plates and allowed to adhere for 24 h. To evaluate the cytotoxicity of TBHP, SH-SY5Y cells were treated by TBHP for 2 h; then the drug was washed out and cells were incubated for 48 h. After adhesion for 24 h, SH-SY5Y cells were treated with noni juice extracts for 48 h, and then the cytotoxicity of noni juice extracts was evaluated. The cell viability was determined using the Cell Counting Kit-8. In brief, 10 μL CCK-8 solution was added to each well of the plate. Then, the plate was incubated at 37°C for an additional 3 h. The absorbance of the WST-8 formazan dye was measured at 450 nm. The half maximal inhibitory concentration (IC_50_)value was calculated from the nonlinear regression analysis.

### Effects of noni juice extracts on SH-SY5Y cells against TBHP-induced toxicity

To investigate the protective effect of noni juice extracts in TBHP-induced cell death, the cells were incubated with DMEM/F12 for 24 h. After incubation with noni juice extracts for 4 h, cells were treated with TBHP for 2 h and then exposed to noni juice extracts for 48 h. Cell viability was measured by means of the CCK-8 assay.

### Measurement of cellular antioxidant enzymes

SH-SY5Y cells were seeded into 10-cm culture dishes for 24 h. After pretreatment of noni juice extracts for 4 h, cells were incubated with TBHP for 2 h and then exposed to noni juice extracts for 48 h. SH-SY5Y cells were collected from culture dishes and sonicated on ice. The solution was centrifuged for 15 min at 4°C to eliminate cell debris, and the supernatant was used in enzyme activity assays. Antioxidant enzyme activities including CAT, GPx, GR, and SOD-1 were measured as described by Heng-Yin Ju et al. (25).

### Reactive oxygen species and mitochondrial membrane potential assays

The method was performed according to the protocol previously published (26). To determine the effect of CF and AF on TBHP-induced reactive oxygen species (ROS) generation, SH-SY5Y cells were seeded into 6-well plates at a density of 3×10^5^ cells/well and allowed to adhere for 24 h. After incubation with CF or AF for 4 h, cells were treated with TBHP for 2 h and then followed by the addition of various concentrations of CF or AF for 48 h. Then the SH-SY5Y cells for the detection of ROS were incubated with 10 μmol/L 2’,7’-dichlorodihydrofluorescein diacetate (H2DCFDA) at 37°C for 30 min in the dark. For the mitochondrial membrane potential assay, the SH-SY5Y cells were incubated with 0.5 mmol/L Rhodamine 123 at 37°C for 30 min in the dark. ROS generation was analyzed by a BD FACSCalibur cytometerwith the CELLQuest program.

### Reverse transcriptase-polymerase chain reaction

The RNA (500 ng) was reverse-transcribed with the Moloney murine leukemia virus reverse transcriptase (Promega Corp., Madison, WI, USA) and oligo (dT)16 primer (Promega) in 20 μL of reaction mixture. Each PCR primer used in this study was as follows (forward and reverse, respectively): heme oxygenase-1 (HO-1), 5'-CTT GGC TGG CTT CCT TAC C-3' and 5'-CAT TGC CTG GAT GTG CTT T-3'; CAT, 5'-CCG ACG AGA TGG CAC ACT TTGACA-3' and 5'-CGC GAG CAC GGT AGG GAC AGT TC-3'; SOD-1, 5'-CCA TCA ATA TGG GGA CAA TACAC-3' and 5'-ACA CGA TCT TCA ATG GAC AC-3'; GAPDH, 5'-AGT GTA GCC CAG GAT GCC CTT-3' and 5'-GCC AAG GTC ATC CAT GAC AAC-3'.

### Western blot analysis

The method was performed according to the protocol previously published (27). The level of β-actin was used as a loading control. The antibodies against superoxide dismutase 1 (SOD-1) (#4266), Nrf2 (#12721), cleaved/total poly(ADP-ribose) polymerase-1 (PARP-1) (#9532), and cleaved caspase-3 (#9661) were obtained from Cell Signaling Technology, Inc. (Danvers, MA, USA). The antibody to β-actin (HC201) was obtained from TransGen Biotech (BeiJing, China).

### Detection of apoptotic cells by annexin V-(fluorescein isothiocyanate, FITC)/PI

SH-SY5Y cells were seeded into six-well plates at a density of 3×10^5^ cells/well and allowed to adhere for 24 h. After incubation with noni juice extracts for 4 h, cells were treated with TBHP for 2 h, followed by the addition of various concentrations of CF for 48 h. To quantify apoptosis, cells were stained with annexin V and PI using an annexin V-FITC/PI apoptosis kit (BD Biosciences, San Jose, CA, USA), following the protocol provided by the manufacturer. The fluorescence intensity was measured using a BD FACSCalibur flow cytometer according to the protocol previously published (28).

### Statistical analysis

Results were expressed as the mean ± SD from at least three independent experiments. One-way ANOVA was used to analyze the variance for the means of multiple groups. Student’s *t*-test was applied for comparing the means of two groups. Statistical analysis was performed using SPSS 17.0 and significant differences were considered at values of *p* < 0.05.

## Results

### Acute oral toxicity

During the 14-day single-dose acute toxicity study, no mortality or clinical signs of morbidity occurred in Sprague-Dawley rats with 15,000 mg/kg BW of noni juice powder. Body weights of animals were unaffected by treatment (Table 1) and macroscopic examination of main organs showed no apparent abnormalities.

**Table 1:** Effect on body weight in rats acutely treated with noni juice

Noni juice powder (mg/kg)	Sex	Number dosed	Body weight (mean μ SD)	

			Day 1	Day 15
0	M	5	196.4μ12.2	274.9μ11.4
0	F	5	193.3μ9.8	230.4μ10.3
15,000	M	5	198.5μ13.3	275.0μ18.5
15,000	F	5	192.0μ11.8	228.1μ9.9

### Bacterial reverse mutation test (Ames test)

The bacterial reverse mutation tests indicated that noni juice powder did not induce mutagenic activity toward any of the *S. typhimurium* strains with or without S9 activation at concentrations up to 5,000 µg/plate (Table 2).

**Table 2:** *Salmonella typhimurium* reverse mutation test for noni juice by the direct-plate incorporation method with and without S9 activation

Noni juice powder (ug/plate)	Number of revertants/plate							

	TA97		TA98		TA100		TA102	
			
	-	+	-	+	-	+	-	+
Experiment 1								
0	119.3μ14.6	116.0μ12.5	35.7μ5.7	40.0μ3.0	141.7μ12.6	144.3μ13.1	267.3μ16	261.3μ8.1
8	122.3μ17.5	121.7μ14.6	36.0μ3.6	38.0μ5.6	147.0μ17.1	145.0μ14.7	266.7μ15.5	265.7μ16.6
40	120.3μ16.3	125.7μ10.6	36.3μ4.5	39.0μ4.6	150.7μ13.3	144.3μ18.5	269.7μ15.9	266.0μ13.1
200	120.0μ10.0	114.7μ15.0	39.3μ3.1	37.0μ5.0	150.3μ20.6	150.3μ13.5	267.7μ18.0	265.3μ15.0
1,000	119.0μ16.8	123.0μ12.2	37.7μ5.7	39.7μ5.0	155.3μ15.5	154.3μ15.0	271.3μ17.2	267.7μ12.3
5,000	120.3μ10.0	125.0μ9.2	37.7μ3.1	38.7μ4.7	156.7μ13.3	154.3μ13.7	269.7μ13.3	163.3μ18.2
Positive control	1,270.0μ81.9	1,296.7μ97.1	1,926.7μ70.2	1,826.7μ94.5	1,326.7μ73.7	1,336.7μ112.4	1,353.3μ138.0	920.0μ60.0
Experiment 2								
0	117.7μ11.6	120.0μ14.0	42.7μ4.5	41.7μ4.2	153.0μ10.8	146.3μ12.7	264.7μ22.0	267.7μ11.6
8	123.3μ10.4	122.7μ13.0	39.0μ4.6	36.0μ5.6	158.3μ14.7	150.7μ17.2	270.0μ11.4	262.3μ8.6
40	124.7μ12.7	120.7μ11.0	36.7μ4.7	37.7μ5.5	163.0μ12.1	152.3μ17.8	263.7μ12.7	270.7μ13.7
200	120.3μ15.6	127.3μ17.2	39.0μ2.6	37.0μ4.0	155.0μ16.1	143.3μ12.3	271.0μ14.5	265.7μ13.9
1,000	124.7μ9.1	126.3μ13.3	39.7μ5.5	38.0μ2.6	160.3μ16.0	146.7μ17.2	271.7μ18.5	270.7μ14.2
5,000	124.0μ10.8	119.3μ17.8	39.3μ4.2	40.7μ4.7	164.3μ12.1	149.7μ13.6	264.3μ18.9	270.7μ19.0
Positive control	1,293.3μ61.1	1,316.7μ96.1	1,873.3μ92.9	1,880.0μ124.9	1,270.0μ117.9	1,313.3μ101.2	1,436.7μ106.0	890.0μ70.0

### TPC, flavonoid, and polysaccharide contents

TPC, flavonoid, and polysaccharide contents of noni juice extracts were measured systematically by spectrophotometric methods (Table 3). CF contained the highest TPC (284 mg of GAE/g CF) and a higher amount of flavonoids (156 mg of RE/g CF). Each gram of EF contained the highest amount of flavonoids (172 mg of RE) and higher amounts of TPC (255 mg of GAE). However, the amount of polysaccharide (584 mg of GE/g AF) contained in AF was more than other fractions.

**Table 3:** TPC, flavonoid, and polysaccharide contents of noni juice extracts

	TPC (mg of GAE/g)	Flavonoids (mg of RE/g)	Polysaccharides (mg of GE/g)
CF	284.84μ5.36	156.32μ10.11	nd
EF	255.66μ8.84	172.07μ6.89	nd
BF	38.73μ1.25	14.0μ0.23	179.34μ8.64
AF	75.45μ4.21	8.64μ0.18	584.64μ12.28

TPC, total phenolic content; GAE, gallic acid equivalents; RE, rutin equivalents; GE, D-glucose equivalents; CF, chloroform fraction; EF, ethyl acetate fraction; BF, n-butanol fraction; AF, aqueous fraction; nd, not detect.

### Cytotoxic effects of noni juice extracts and TBHP on SH-SY5Y cells

**Fig. 1:**
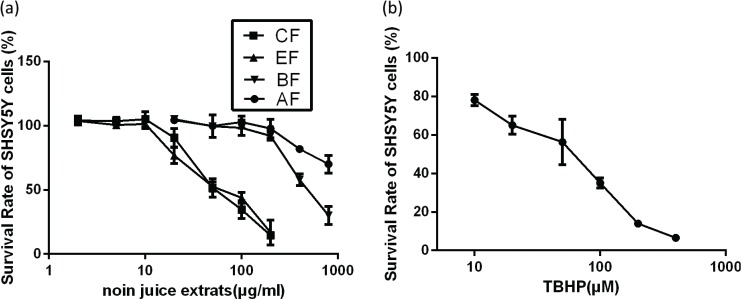
Survival of SH-SY5Y cells treated with noni juice extracts and TBHP. (a) Survival of SH-SY5Y cells that were treated with noni juice extracts for 48 h. (b) Survival of SH-SY5Y cells that were treated with TBHP for 2 h. Data represent the mean ± SD of three independent experiments. TBHP, tert-Butyl hydroperoxide.

To determine doses of noni juice extracts that could be used without adversely affecting cell viability, the latter was determined using the CCK-8 assay and plotted on the survival curves. The IC_50_ values of CF and EF for SH-SY5Y cells were 65.13 and 65.72 μg/mL, respectively (Fig. 1a). No cytotoxicity or changes in cell morphology were observed with 10 μg/mL CF or EF. The IC_50_ values for BF and AF were both greater than 0.5 mg/mL. In order to evaluate the cytotoxicity of TBHP, SH-SY5Y cells were treated with different concentrations of TBHP (5–500 μM) and a dose-dependent cell death was observed (Fig. 1b). Concentrations of TBHP ≥50 μM dramatically decreased cell viability and cell death reached 40% after 50 μM TBHP treatment.

### CF and AF confer protection on SH-SY5Y cells against TBHP-induced toxicity

**Fig. 2:**
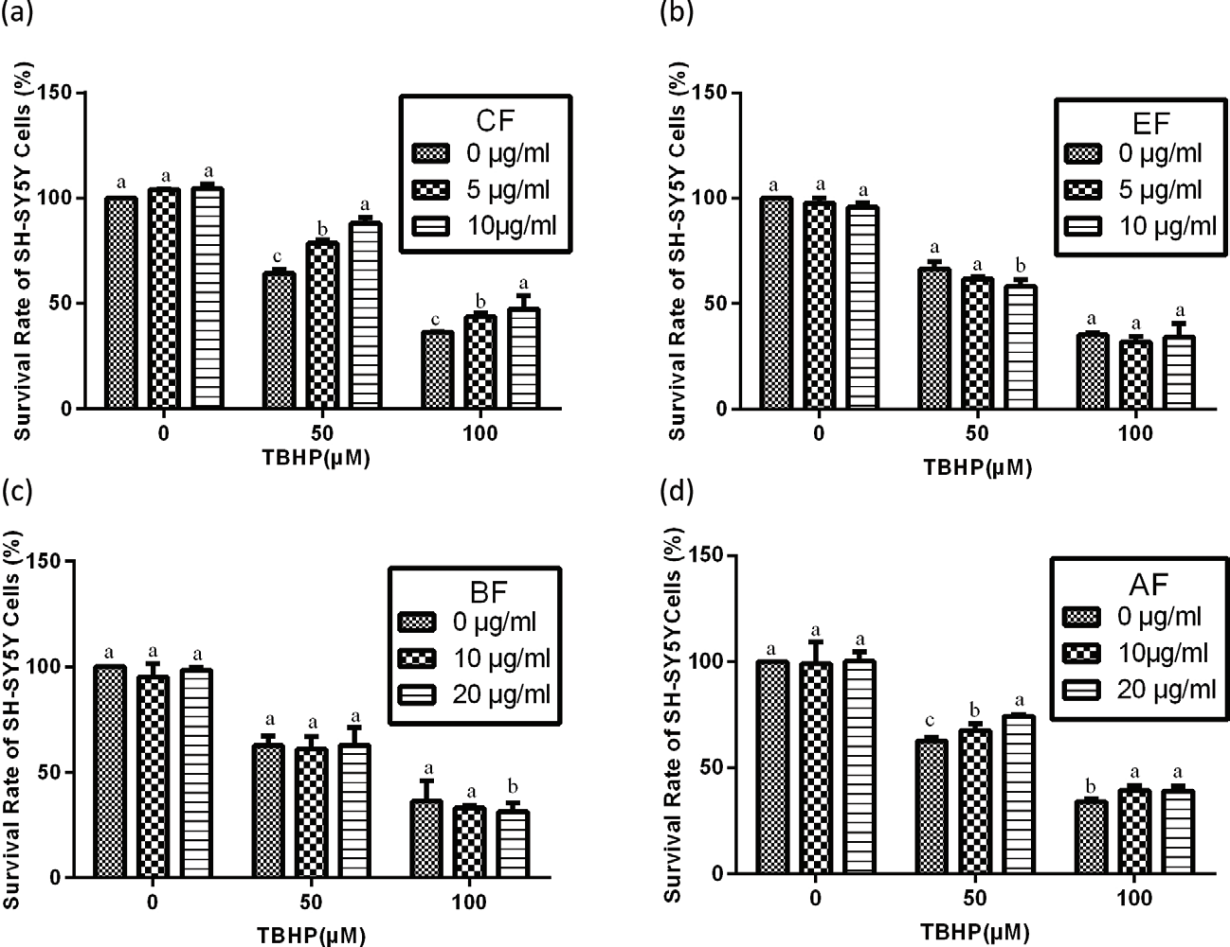
Effects of noni juice extracts on TBHP-treated SH-SY5Y cells. After incubation with noni juice extracts for 4 h, SH-SY5Y cells were treated with TBHP for 2 h and then exposed to noni juice extracts for 48 h. Data are expressed as mean ± SD (*n* = 3). ^a–c^The bars with different letters represent significant difference (*p* < 0.05), compared with each control group.

In the present study, we evaluated the protective effect of noni juice extracts against TBHP challenge by CCK-8 assay. To determine the protective effects of noni juice extracts against TBHP-induced cytotoxicity, SH-SY5Y cells were preincubated with noni juice extracts for 4 h, treated with TBHP for 2 h, followed by treatment with noni juice extracts for 48 h. With 50 μM TBHP, only 60.6% of cells were viable as compared to the control group. However, as shown in Fig. 2, it was noteworthy that TBHP-induced cell death was significantly ameliorated by CF and AF treatments. Treatment with 10 μg/mL CF markedly improved cell survival up to 87.8%, and 20 μg/mL AF reestablished cell viability to 76.4%. However, neither EF nor BF had any significant effect on cell viability.

### Effects of noni juice extracts on antioxidant enzymes activity in TBHP-treated SH-SY5Y cells

In order to investigate whether antioxidant enzymes are involved in the removal of free radicals by noni extracts, SOD-1, CAT, GPx, and GR activities in SH-SY5Y cells were measured by the spectrophotometric degradation method. As shown in Table 4, oxidative damage induced by TBHP remarkably reduced the activity of antioxidant enzymes, while noni juice extracts pretreatment restored their activities almost similar to the control group, indicating that active ingredients in noni juice extracts have antioxidant defense ability (Table 4). Moreover, greater restoring capacity for antioxidant enzymes was observed in CF and AF groups, which was consistent with the result that CF and AF treatment conferred protection on SH-SY5Y cells against TBHP-induced toxicity. CF exhibited the best restoring capacity for antioxidant enzymes. It is speculated that higher activity of antioxidant enzymes could attenuate ROS-induced cell damage more efficiently.

**Table 4:** Effects of noni juice extracts on SOD-1, CAT, GPx, and GR activity in SH-SY5Y cells exposed to 50 μM TBHP

Samples	SOD-1 (U/mg)	Catalase (U/mg)	GPx (mU/mg)	GR (mU/mg)
Control	43.2μ2.93**	1.84μ0.16*	37.3μ2.58**	8.89μ0.68**
50 μM TBHP	18.7μ3.79	1.01μ0.08	23.0μ2.08	3.45μ0.58
5 μg/mL CF+ TBHP	45.5μ2.99**	2.16μ0.17**	40.8μ1.82**	9.94μ0.67**
5 μg/mL EF+ TBHP	34.1μ2.00**	1.61μ0.10*	30.2μ2.42*	8.9μ0.91**
10 μg/mL BF+ TBHP	37.2μ2.38**	2.26μ0.05**	36.2μ3.48**	4.97μ0.89*
10 μg/mL AF+ TBHP	43.0μ3.79**	2.07μ0.06**	40.1μ1.75**	9.7μ1.31**

After incubation with noni juice extracts for 4 h, SH-SY5Y cells were treated with TBHP for 2h and then exposed to noni juice extracts for 48 h. Data are expressed as mean ± SD (*n* = 3). *p < 0.05 and **p < 0.01 as compared to TBHP-treated cells.

### CF and AF inhibits TBHP-induced ROS generation

**Fig. 3:**
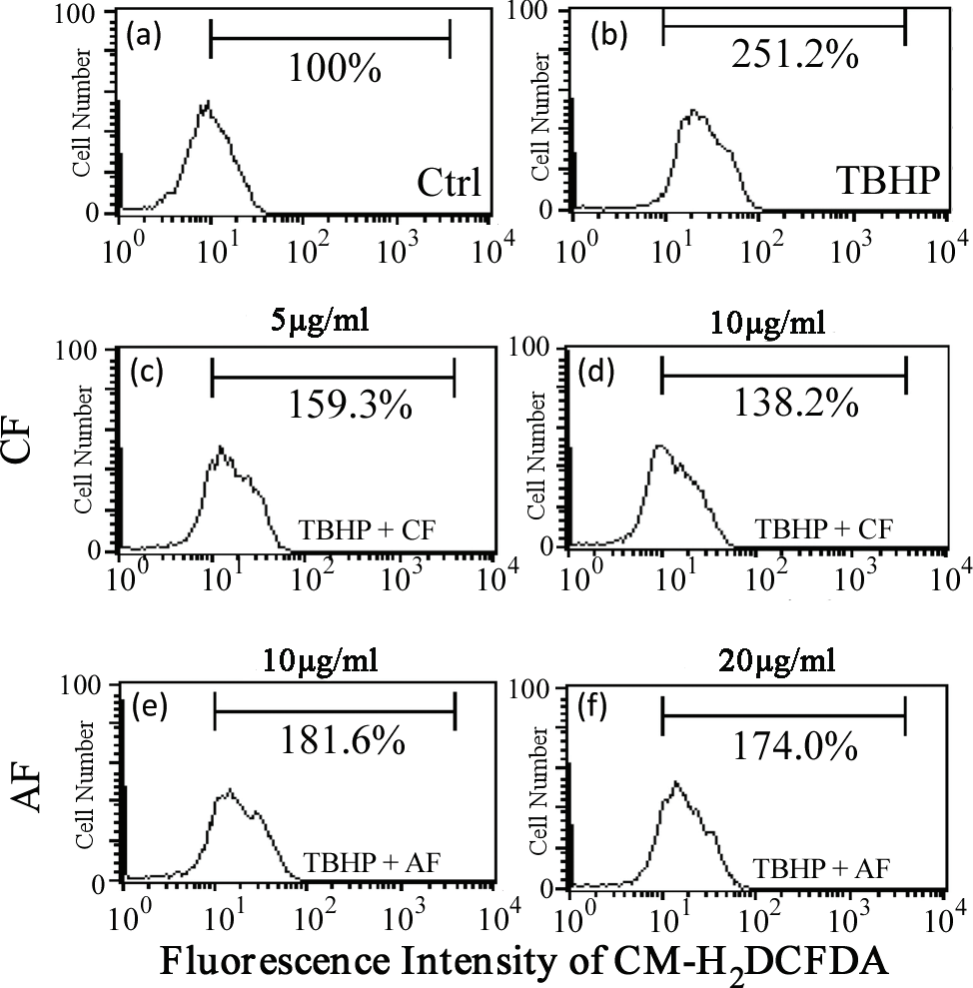
Effect of CF and AF on TBHP-induced ROS generation. SH-SY5Y cells were treated with (a) 0, (b) 50 μM TBHP, (c) 5 μg/mL CF + 50 μM TBHP, (d) 10 μg/mL CF + 50 μM TBHP, (e) 10 μg/mL AF + 50 μM TBHP, (f) 20 μg/mL AF + 50 μM TBHP. After treatment, the cells were stained with H2DCFDAfor 30 min and then analyzed by flow cytometry. CF, chloroform fraction; AF, aqueous fraction; ROS, reactive oxygen species.

Further, the effects of CF and AF on TBHP-induced ROS generation were investigated. As shown in Fig. 3, compared with the control group, TBHP treatment markedly increased the fluorescence intensity of SH-SY5Y cells to 251.2%. However, 5 μg/mL CF and 10 μg/mL AF significantly decreased the fluorescence intensity to 159.3 and 181.6%, respectively. The results indicated that CF and AF could eliminate endogenous ROS in SH-SY5Y cells treated with TBHP efficiently.

### Inhibitory effects of CF and AF on TBHP-induced disruption of mitochondrial membrane potential (MMP)

**Fig. 4:**
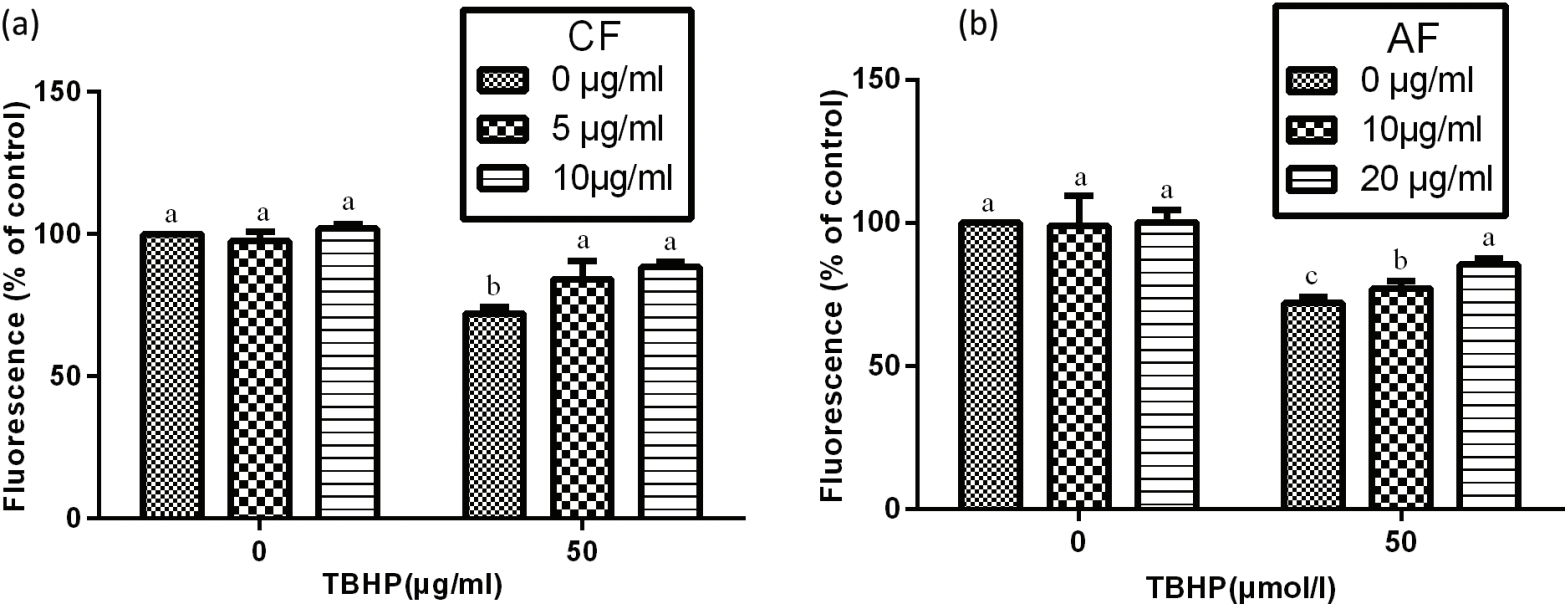
Estimation of mitochondrial membrane potential in SHSY5Y cells. After incubation with CF or AF for 4 h, SH-SY5Y cells were treated with TBHP for 2 h and then exposed to CF or AF for 48 h. After staining with Rhodamine 123 for 30 min, the cells were assayed with a FACSCalibur cytometer. Data are expressed as mean ± SD (*n* = 3). ^a–c^The bars with different letters represent significant difference (*p* < 0.05), compared with each control group.

In order to examine whether the protection of CF and AF on SH-SY5Y cells against TBHP-induced toxicity involves the MMP pathway, measurement of MMP was carried out. Data showed that treatment with TBHP resulted in a marked decrease of MMP to 70.3%, which demonstrated that TBHP induced the mitochondrial damage via depolarization of MMP. However, pretreatment with 5 μg/mL CF and 10 μg/mL AF restored the MMP to 85.5 and 80.2%, respectively. These results indicated that CF and AF potentially suppressed the depolarization of MMP and partially showed the protective effects against TBHP-induced toxicity (Fig. 4).

### Effects of CF and AF on HO-1, CAT, and SOD-1 gene expression

**Fig. 5:**
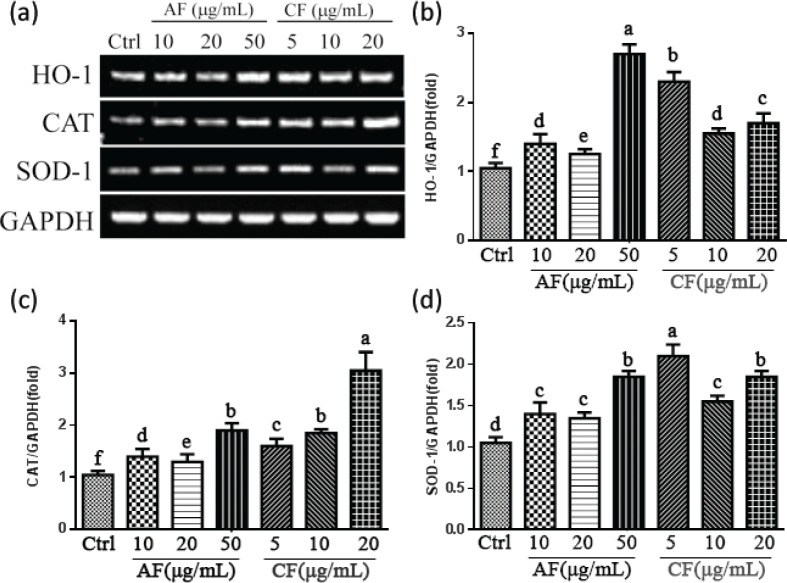
Effects of CF and AF on HO-1, CAT, and SOD-1 gene expression. (a) SH-SY5Y cells were pretreated with AF and CF for 24 h. Gene expression was analyzed by RT-PCR. (b) Quantification of HO-1 gene expression. (c) Quantification of CAT gene expression. (d) Quantification of SOD-1 gene expression. ^a–f^The bars with different letters represent significant difference (*p* < 0.05). HO-1, heme oxygenase-1; CAT, catalase; SOD-1, superoxide dismutase-1.

To explore the molecular mechanism of CF and AF protection on SH-SY5Y cells, gene expression of antioxidants and Phase II detoxifying enzymes such as HO-1, CAT, and SOD-1 were analyzed by Reverse Transcription-Polymerase Chain Reaction (RT-PCR). As shown in Fig. 5a, CF and AF treatment increased the expression of HO-1, CAT, and SOD-1 gene expression. Compared to the control group, treatment with 5 μg/mL CF upregulated HO-1, CAT, and SOD-1 gene expression 2.30-fold, 1.65-fold, and 2.10-fold, respectively, while 10 μg/mL AF upregulated HO-1, CAT, and SOD-1 gene expression 1.28 times, 1.35 times, and 1.47 times, respectively (Fig. 5b through d).

### Effect of CF and AF on Nrf2 nuclear translocation

**Fig. 6:**
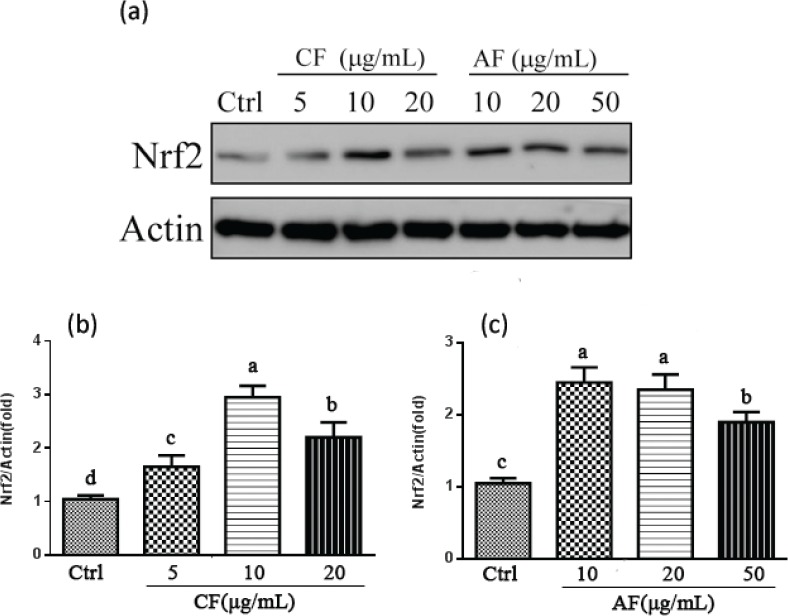
Effect of CF and AF on the nuclear translocation of Nrf2. (a) SH-SY5Y cells were pretreated with CF and AF for 24 h. Nuclear extracts were prepared and analyzed by Western blot. (b) Quantification of Nrf2 protein expression pretreated with CF. (c) Quantification of Nrf2 protein expression pretreated with AF. ^a–d^The bars with different letters represent significant difference (*p* < 0.05).

The activation of Nrf2/ antioxidant response element (ARE) pathway is well known to confer resistance of cells to oxidative stress. In order to understand whether the upregulation of antioxidant enzymes and Phase II detoxification enzymes by CF and AF were related to activation of Nrf2, the transactivation of Nrf2 in nuclear fractions was examined by Western blotting. As illustrated in Fig. 6a, CF and AF treatment obviously increased Nrf2 accumulation compared to the control group. A 3.16-fold increase in the nucleus Nrf2 was observed in the 10 μg/mL CF treatment group by comparison with 2.35-fold in the 10 μg/mL AF group (Fig. 6b). It is likely that the Nrf2 signaling pathway is an important neuroprotective mechanism of CF and AF against TBHP-induced oxidative damage in SH-SY5Y cells.

### CF inhibited TBHP-induced apoptotic cell death

**Fig. 7:**
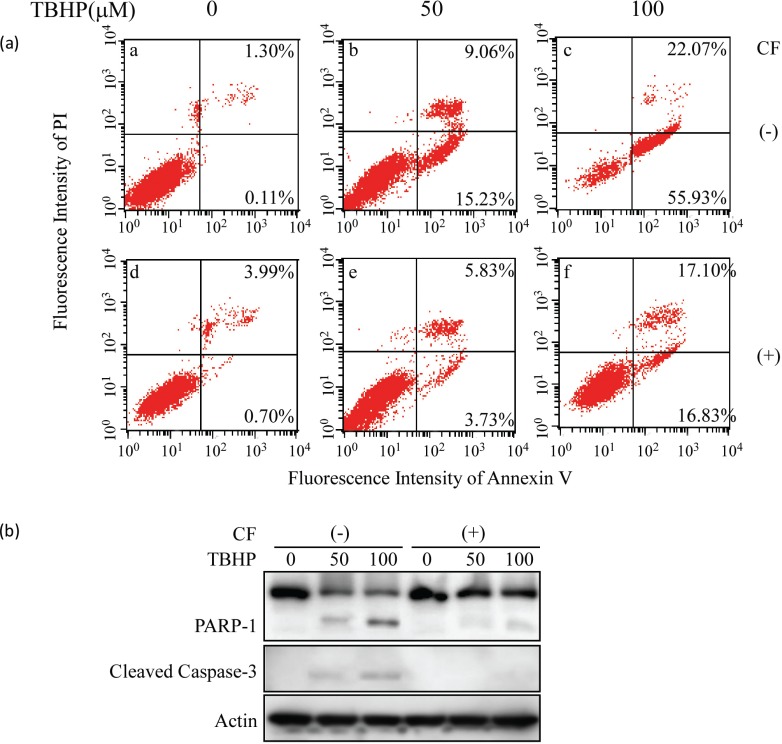
Effect of CF on TBHP-induced apoptosis in SH-SY5Y cells. (a) Apoptotic cells were detected by annexin V and PI double staining and analyzed by flow cytometry. (b) Western blot analysis of the status of PARP-1 and cleaved caspase-3. β-actin was used as a loading control. A representative result of three independent experiments is shown. PARP-1, poly(ADP-ribose) polymerase-1; PI, propidium iodide.

To explore whether CF and AF conferring protection on SH-SY5Y cells against TBHP-induced toxicity was associated with apoptosis, the double-staining method using FITC-labeled annexin V and PI was performed. CF was chosen to investigate the mechanism of its protection on TBHP-induced cell apoptosis. As shown in Fig. 7a, the cell apoptosis rate of the control group was 1.41% and that of the 50 μM and 100 μM TBHP groups was obviously increased to 24.29 and 78.0%. However, 10 μg/mL CF treatment significantly reduced the TBHP-induced cell apoptosis rate of the 50 μM and 100 μM TBHP groups, respectively, to only 9.56 and 33.93%. These results indicated that CF exhibited protective effects against oxidative stress-induced apoptosis.

In the present study, we examined whether THBP-induced cell death was mediated through native caspase-3 cleavage and PARP-1 degradation and further explored the molecular mechanisms of the neuroprotective effect of CF on THBP-induced apoptosis. As shown in Fig. 7b, cleaved caspase-3 protein and degradation of PARP-1 were undetected in the control group but appeared after exposure to TBHP. However, CF significantly prevented the activation of caspase-3 and cleavage of PARP-1, indicating that CF could reverse the TBHP-induced apoptotic cell death efficiently. These results demonstrated that CF exhibited protective effects against TBHP-induced SH-SY5Y cells apoptosis through preventing activation of caspase-3.

## Discussion

Although noni juice is well known as an antioxidant, the anti-oxidation effect and molecular mechanism of its extracts against oxidative damage in neurons are at present unclear. In this study, SH-SY5Y cells with oxidative damage induced by TBHP were used as a model to explore the antioxidant and neuroprotective effects of noni juice extracts and reveal the underlying mechanism.

The acute oral toxicity and genotoxicity potential of noni juice were investigated. Noni juice did not induce mortality, clinical signs of morbidity, or physiologic abnormalities in Sprague-Dawley rats at a dose of 15,000 mg/kg BW and was also nongenotoxic at concentrations up to 5,000 µg/plate. The results indicated that the maximum tolerated dose of noni juice was > 215.38 mL/kg BW in rats. These results suggest low toxicity of noni juice in acute toxicity studies.

Phenolic compounds and Polysaccharides of valuable plants for health care and medicine have been proven to be the main classes of natural antioxidants and correlated with their antioxidant activities (29–32). Our present study showed that CF contained the highest amount of TPC, while AF had a higher content of polysaccharides than other fractions. Recent reports also indicate that TPC, flavonoids, and polysaccharides are the bioactive compounds present in noni juice, which are responsible for its antioxidant and other pharmacological properties (15–17).

Previous studies revealed that several herbal extracts could inhibit TBHP-induced cell loss efficiently (33, 34). In this study, pretreatment with CF and AF significantly prevented cell loss in a dose-dependent manner. However, EF and BF had no ability to improve cell viability. These results indicated that CF and AF protected SH-SY5Y cells in the presence of TBHP-induced oxidative stress. The high TPC and flavonoid content of CF may account for its antioxidant and neuroprotection potential, while AF may be rich in polysaccharides.

ROS is a major pathological factor that can lead to many serious diseases, including neurodegeneration and cardiovascular diseases. Intracellular ROS production disrupts redox balance or oxidative stress not only in relation to cell proliferation and signal transduction but also to inducing apoptosis (35, 36). Because ROS serves as an initiator of TBHP-induced toxicity, we measured intracellular ROS levels subsequently. Our results showed that CF and AF were able to inhibit TBHP-induced ROS generation, which indicated that the neuroprotective effects of CF and AF may be mediated by blocking ROS overproduction. CF reduced the apoptosis rate caused by TBHP, indicating that changes in ROS levels are essential for CF protection against TBHP-induced cell apoptosis. The results are similar to previous findings that demonstrate the ROS scavenging activity of *Cyperus* extract against H_2_O_2_-induced neuronal stress (37).

Antioxidant enzymes such as CAT, GPx, SOD-1, and GR play important roles in detoxifying ROS and maintaining redox status. Compelling evidence demonstrates that the activities of antioxidant enzymes decrease significantly in many neurodegenerative diseases (38). Perhaps the neuroprotective effects of noni juice extracts were related to antioxidant enzyme action. In order to further confirm the participation of antioxidant status, we examined their activities. CF and AF inhibited TBHP-induced reduction of SOD-1, CAT, GPx, and GR levels. The results demonstrated that the neuroprotective effects of CF and AF may partly be mediated through enhancing the antioxidant enzyme activity. Previous reports also showed that the neuroprotective effects of natural plants are associated with activation of antioxidant enzyme activity (10, 25).

Nrf2 is a major regulator for ARE-driven antioxidant and Phase II detoxifying enzyme expressions, such as NAD(P)H quinone oxidoreductase 1 (NQO1), HO-1, CAT, and SOD-1. Increasing studies reveal that these antioxidant enzymes are regulated by activation of Nrf2 and play an important role in oxidative stress-induced neuronal injury (39–42). Our results showed that CF and AF treatment increased the nuclear translocation of Nrf2, which was also associated with elevated antioxidative enzyme expressions of HO-1, CAT, and SOD-1. In addition, induction of HO-1 expression was higher than that of SOD-1 and CAT, indicating that HO-1 may be a major factor exerting neuroprotective effects. It is hypothesized that promoting antioxidants and Phase II detoxifying enzymes via activating Nrf2 nuclear translocation might be responsible for CF and AF protecting SH-SY5Y cells from TBHP-induced oxidative damage.

A collapse of MMP is associated with several models of apoptosis and used to assess stress-induced apoptotic cell damage (43–45). Mitochondrial damage has been observed in Parkinson’s disease by inhibition of Complex I activity, which results in mitochondrial impairment. Our results are in agreement with other reports that oxidative stress induced by TBHP impairs the mitochondrial membrane, resulting in the depolarization of MMP. CF and AF potentially restrained the depolarization of MMP induced by TBHP. In this study, we further explored the possible role of CF in TBHP-induced mitochondrial apoptosis.

TBHP-induced apoptosis mainly occurs via the apoptotic caspase pathway; apoptosis is induced by initiating mitochondrial dysfunction (46). Caspases are important mediators of cell death through the cleavage of many substrates. Caspase-3 is an executioner for the death program in response to various stressors. Activated caspase-3 catalyzes the degradation of PARP-1, which is an important ribozyme in DNA repair, cell proliferation, apoptosis, and transcription (47). The results in Fig. 7b show that CF protected TBHP-induced apoptosis via preventing caspase-3 activation.

In summary, this study demonstrated the antioxidant potential and molecular mechanisms involved in the neuroprotective effects of CF and AF. Our findings suggest that CF and AF can provide neuroprotection for SH-SY5Y cells against TBHP-induced oxidative damage and apoptosis through improving the antioxidant status, maintaining the mitochondrial membrane integrity, and regulating the apoptotic markers. Moreover, CF and AF could elevate antioxidant and Phase II detoxifying enzyme expression through activating the nuclear translocation of Nrf2, which may be the underlying molecular mechanism for protecting SH-SY5Y cells from TBHP-induced oxidative damage. It can be concluded that CF and AF are potential candidates for preventing neuronal-associated disorders mediated by oxidative stress. However, it is necessary to further explore the derivatized compounds in CF and AF with better neuroprotective activity, which should be investigated as a natural remedy for neurodegenerative disorders.
